# Glycogen metabolism and the homeostatic regulation of sleep

**DOI:** 10.1007/s11011-014-9629-x

**Published:** 2014-11-16

**Authors:** Jean-Marie Petit, Sophie Burlet-Godinot, Pierre J. Magistretti, Igor Allaman

**Affiliations:** 1 Laboratory of Neuroenergetics and Cellular Dynamics, Brain Mind Institute, Ecole Polytechnique Fédérale de Lausanne (EPFL), Lausanne, 1015 Switzerland; 2 Division of Biological and Environmental Sciences and Engineering, KAUST, Thuwal, KSA Saudi Arabia; 3 Center for Psychiatric Neuroscience, Department of Psychiatry, CHUV, 1008 Prilly, Switzerland

**Keywords:** Glia, PTG, Glucocorticoïds, Energy metabolism, Noradrenaline, Brain

## Abstract

In 1995 Benington and Heller formulated an energy hypothesis of sleep centered on a key role of glycogen. It was postulated that a major function of sleep is to replenish glycogen stores in the brain that have been depleted during wakefulness which is associated to an increased energy demand. Astrocytic glycogen depletion participates to an increase of extracellular adenosine release which influences sleep homeostasis. Here, we will review some evidence obtained by studies addressing the question of a key role played by glycogen metabolism in sleep regulation as proposed by this hypothesis or by an alternative hypothesis named “glycogenetic” hypothesis as well as the importance of the confounding effect of glucocorticoïds. Even though actual collected data argue in favor of a role of sleep in brain energy balance-homeostasis, they do not support a critical and direct involvement of glycogen metabolism on sleep regulation. For instance, glycogen levels during the sleep-wake cycle are driven by different physiological signals and therefore appear more as a marker-integrator of brain energy status than a direct regulator of sleep homeostasis. In support of this we provide evidence that blockade of glycogen mobilization does not induce more sleep episodes during the active period while locomotor activity is reduced. These observations do not invalidate the energy hypothesis of sleep but indicate that underlying cellular mechanisms are more complex than postulated by Benington and Heller.

While sleep has been investigated over decades, the fundamental question of why we need to sleep is still unanswered. More specifically, the physiological function(s) fulfilled by sleep remain(s) elusive. Among the formulated hypotheses is the necessity of sleep to reestablish brain energy stores that are altered during wakefulness. The main postulate behind this theory is that energy stores are diminished during the metabolically active waking period and need to be restored during sleep. Based on this, Benington and Heller formulated almost 20 years ago an energy hypothesis of sleep proposing glycogen and adenosine (Ade) as key regulators of sleep homeostasis (Benington and Heller 1995). This model suggests that glycogen stores depletion occurs during waking leading to Ade formation in the extracellular space, which in turn acts as a promoting agent of sleepiness influencing sleep homeostasis. As glycogen is exclusively found in astrocytes, a glial cell type, in the central nervous system (CNS), this points to a major role of astrocyte functions (in particular with regard to brain energy homeostasis) in sleep regulation.

Since the formulation of Benington and Heller hypothesis, numerous studies have been undertaken to validate, or not, the importance of glycogen store depletion and replenishment as a key regulator of sleep. This review will first introduce basic concepts of brain glycogen metabolism and of the structure and regulation of sleep. Then, major findings related to the involvement of glycogen in sleep will be discussed, focusing on:

- Benington and Heller hypothesis (BHH)
- “Glycogenetic” hypothesis
- A synthesis of experimental data
- Effect of direct modulation of glycogen levels on sleep
- Confounding effects of glucocorticoïds (GCs)

## Brain glycogen metabolism

The precise functions of brain glycogen are still not fully characterized. Nevertheless, it has been shown that glycogen reserves are consumed during failure of energy supply and strong evidence suggests that glycogen mobilization is tightly coupled to neuronal activity and could provide additional energy substrates for neurones during period of activity. For instance, glycogen accumulation is observed in conditions of decreased neuronal activity such as phenobarbital anaesthesia (Phelps 1972), hibernation (Swanson 1992) or during slow wave sleep (SWS) (Karnovsky et al. 1983). In contrast, cerebral glycogen breakdown is observed following sensory stimulation (Dienel et al. 2007; Swanson et al. 1992). Moreover, the greatest accumulation of glycogen has been reported in areas of high synaptic density (Koizumi and Shiraishi 1970; Koizumi and Shiraishi 1971; Phelps 1972), further supporting the idea that glycogen may be involved in neuronal activity. Experiments also demonstrated that glycogen is mobilized to support energy needs of axons upon action potential propagation in an optic nerve preparation (Brown et al. 2003; Brown et al. 2004).

**Fig. 1:**
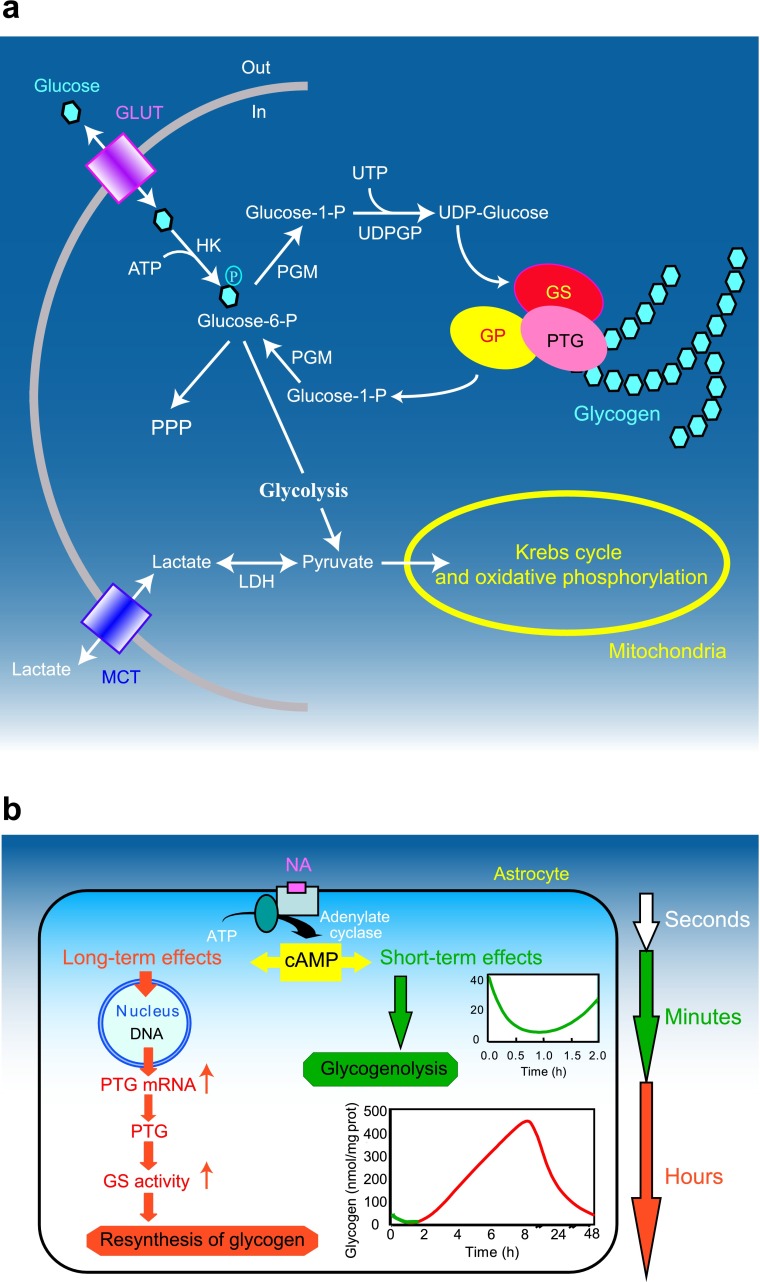
Gylcogen metabolism in astrocytes. **a** Schematic representation of glucose metabolism. Glucose enters cells trough glucose transporters (GLUT) and is phosphorylated by hexokinase (HK) to produce glucose-6-phosphate. Glucose 6-phosphate can be processed into different metabolic pathways. It can be metabolized through the pentose phosphate pathway (PPP) or through glycolysis giving rise to pyruvate production. Pyruvate can enter mitochodria where it is metabolized through the Krebs cycle and oxidative phosphorylation. Alternatively, pyruvate can be reduced to lactate by lactate dehydrogenase (LDH) and be released in the extracellular space through monocarboxylate transporters (MCT). Glucose-6-phosphate can also be used to store glucosyl units as glycogen. As a first step glucose-6-phosphate is converted to UDP-glucose through the action of phosphoglucomutase (PGM) and UDP-glucose pyrophosphorylase (UDPGP). UDP-glucose is then incorporated into glycogen by glycogen synthase (GS). Protein targeting to glycogen (PTG) is a specific glycogen-binding G-subunit, which is responsible for the targeting of protein phosphatase 1 to glycogen. Through this action, PTG promotes GS dephosphorylation and activation therefore favouring glycogen synthesis. In case of energy needs, glycogen can be broken down by glycogen phosphorylase (GP) to produce glucose-1-phosphate that is converted back to glucose-6-phosphate through the action of PGM. Adapted from (Allaman 2009) with permission. **b** Differential modulation of glycogen metabolism in cultured astrocytes by noradrenaline (NA). Activation of cAMP-dependent intracellular signalling by NA results in a short term (seconds to minutes, in *green*) glycogenolysis and in a delayed (hours, in *red*) glycogen resynthesis. This long-term response requires induction of gene expression and is accompanied by stimulation of mRNA expression of protein targeting to glycogen (PTG), a member of the glycogen-targeting subunits of protein phosphatase 1, and by the activation of glycogen synthase (see text for details). Adapted from (Magistretti 2006) with permission

Most of our knowledge about glycogen metabolism originates from studies conducted in peripheral tissues but are common for all tissues (see e.g. Bollen et al. 1998; Jensen and Richter 2012; Roach et al. 2012) (Fig. 1a). Uridine diphosphate glucose (UDP-glucose) is the source of all glucosyl residues added to glycogen. UDP-glucose is formed from the conversion of glucose-6-phosphate to glucose-1-phosphate by phosphoglucomutase. The first step in the biogenesis of glycogen is the autocatalytic attachment of glucosyl units to a single tyrosine residue of glycogenin. Elongation of this “primed” glycogenin is performed by the concerted action of glycogen synthase (GS) and branching enzyme. Glycogen synthase elongates the glycogen chain whereas branching enzyme produces new branches, creating a mature glycogen granule. In contrast to glycogen synthesis, glycogenolysis is a process that does not require energy. It is not a reverse reaction of glycogen synthesis, but implies the concerted action of glycogen phosphorylase (GP) and the bi-functional debranching enzyme releasing glucose residue of glycogen granules as glucose-1-phosphate. Finally, glucose-1-phosphate is converted by phosphoglucomutase to glucose-6-phosphate which is (in the brain) the final product of glycogen degradation. Glucose-6-phosphate can then re-enter glycolysis or the pentose phosphate pathway (Fig. 1a).

Glycogen is a highly dynamic pool of glucose, the metabolism of which is under complex regulation involving various allosteric factors as well as covalent modifications and compartmentalization of key enzymes (for reviews see Bollen et al. 1998; Brown 2004). In general, activation of glycogen synthesis results in the inhibition of the degradative pathway, and vice versa. Glycogen synthase and GP represent two central elements of this regulation. Glycogen synthase is tightly controlled by the reversible phosphorylation of multiple serine residues by various protein kinases. Generally, phosphorylation is associated with an inactivation of the enzyme (conversion of the active a-form to the b-form). Like GS, GP is regulated by phosphorylation. Glycogen phosphorylase is converted from the inactive b-form to the active a-form through phosphorylation by phosphorylase kinase. As described, kinase-catalyzed phosphorylation of both enzymes leads to glycogenolysis (by activation of GP and inactivation of GS) whereas dephosphorylation favors glycogenesis.

Protein phosphatase-1 (PP-1) is the enzyme responsible for dephosphorylation of both GS and GP (Newgard et al. 2000). In order to be active, PP-1 must be bound to glycogen. This is accomplished by specific glycogen-binding G-subunits, which are responsible for targeting PP-1 to glycogen. Among them, two are expressed in the brain: the human PPP1R3C (and its mouse homologue Protein Targeting to Glycogen, PTG) (Doherty et al. 1996; Printen et al. 1997) and PPP1R6 (Armstrong et al. 1997). In addition to its capacity to target PP-1 to glycogen, PTG is also able to bind several of the enzymes involved in glycogen metabolism including GS, GP a-form and phosphorylase kinase (Brady et al. 1997; Printen et al. 1997). Based on these observations, it has been suggested that PTG could act as a molecular scaffold protein assembling the different enzymes necessary for glycogen metabolism (Brady et al. 1997; Mastick et al. 1998).

At the cellular level, glycogen is mainly located in astrocytes in the CNS (Brown 2004; Cataldo and Broadwell 1986; Magistretti et al. 1993). The cellular localization of GS and GP correlates with the presence of glycogen. Both brain and muscle isozymes of GP are located predominantly in astrocytes (Ignacio et al. 1990; Pfeiffer-Guglielmi et al. 2003).

As in peripheral organs, brain glycogen levels are also under the tight control of numerous neuro-humoral factors. Thus, insulin and insulin-like growth factor I increase glycogen content of astrocytes in culture (Dringen and Hamprecht 1992; Hamai et al. 1999). Moreover, various neuroactive substances possess glycogenolytic properties in vitro (for review see Magistretti et al. 1993). In particular, a glycogenolytic effect of potassium, vasoactive intestinal peptide (VIP), Ade and noradrenaline (NA) have been demonstrated in mouse cerebral cortical slices as well as in primary cultures of cortical astrocytes (Hof et al. 1988; Magistretti et al. 1981; Quach et al. 1978; Sorg and Magistretti 1991).

In addition to their rapid glycogenolytic effect, NA, VIP and Ade lead to massive glycogen resynthesis in cultured astrocytes, which takes place over several hours (Allaman et al. 2003; Sorg and Magistretti 1992). It was determined that this long-term dynamic regulation of glycogen in astrocytes requires an activation of transcription and the synthesis of new protein(s). This process is mediated by cAMP-dependent mechanisms, involves the family of transcription factors CCAAT/enhancer binding protein (C/EBP) and results in the induction of PTG expression and of GS activity (Allaman et al. 2000; Cardinaux and Magistretti 1996; Sorg and Magistretti 1992) (Fig. 1b). Interestingly, overexpression of PTG in CHO cells (Printen et al. 1997), in cultured rat hepatocytes (Berman et al. 1998) or in cultured mouse cortical astrocytes (unpublished data) leads to an increase in basal glycogen levels without the need of other stimuli. It was further suggested that increased expression of PTG might maintain the cell in a glycogenic mode (Berman et al. 1998). This set of observations finally suggests that in astrocytes, the induction of PTG alone might be sufficient to account for the massive glycogen resynthesis triggered by neuromodulators such as NA, VIP or Ade.

## Structure and regulation of sleep

**Fig. 2:**
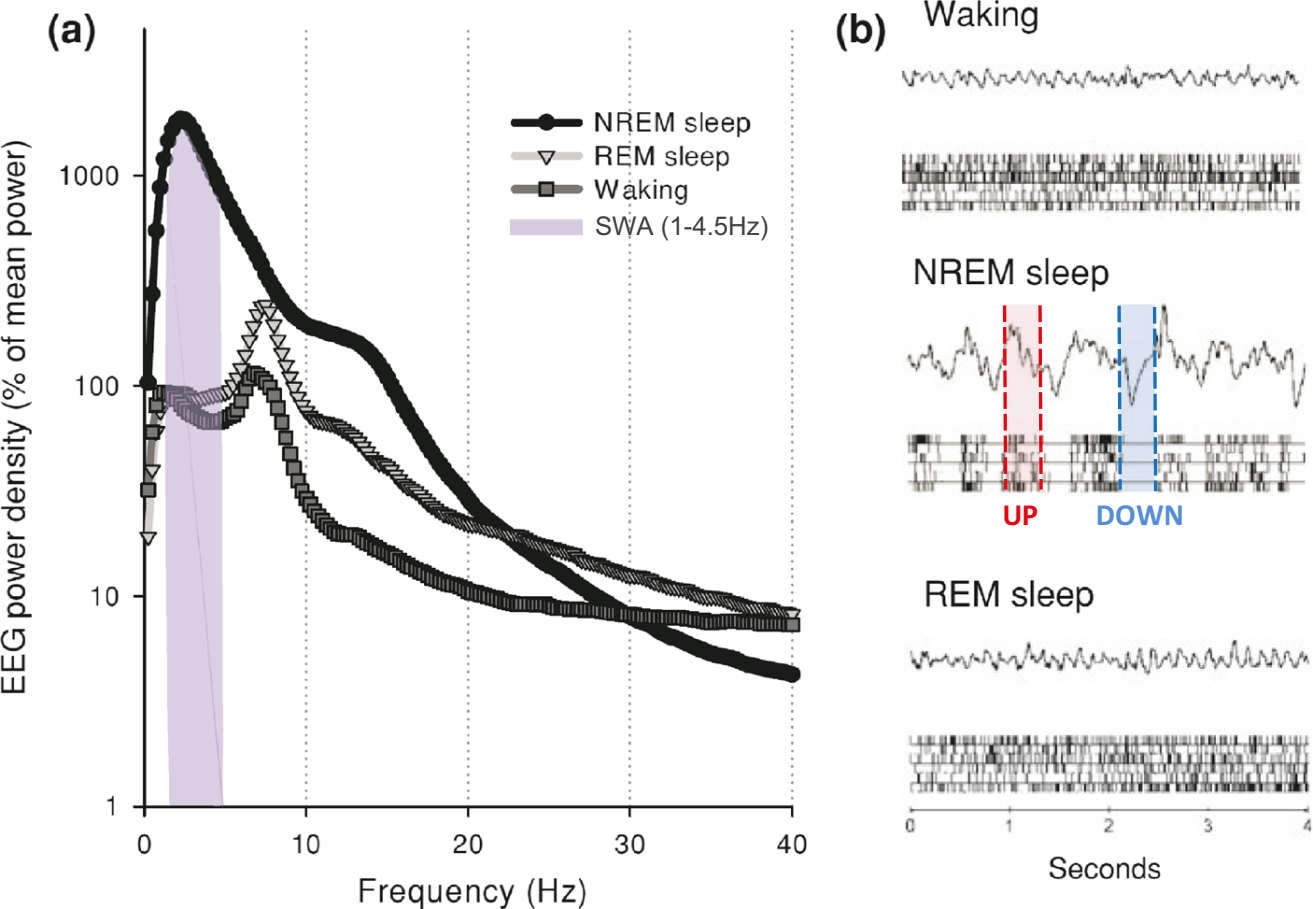
Sleep architecture. **a** Twenty four hour EEG power spectra in NREM sleep (SWS), REM sleep (PS) and waking in one representative adult male rat (Wistar Kyoto strain). Slow Wave Activity (SWA) frequency spectrum (1–4.5 Hz) is highlighted in grey. **b** EEG signals and corresponding cortical multiunit activity (raster plots below; each bar is a spike) representative of the three vigilance states. Note the fast irregular pattern of cortical firing in waking and REM sleep, and regular occurrence of generalized neuronal silence, corresponding to EEG slow waves, in NREM sleep. UP (*red*) and DOWN (*blue*) states during NREM sleep are highlighted. Adapted from (Vyazovskiy and Faraguna 2014) with permission

Behavioral and neuronal correlates of sleep including locomotor inactivity, specific body posture, high threshold of sensory reactivity and decrease in global neuronal firing rate, are shared by numerous animal species from insects to mammals (Campbell and Tobler 1984). This has justified the use of drosophila and zebrafish as well as rodents as experimental models in sleep studies (Cirelli and Tononi 2008; Zimmerman et al. 2008). Using parallel recordings of electroencephalogram (EEG) and electromyogram (EMG), sleep in mammals and birds can be divided in two main stages: Slow Wave Sleep (SWS) also called Non-Rapid Eye Movement (NREM) sleep and Paradoxical Sleep (PS) equivalent to Rapid Eye Movement (REM) sleep. The three vigilance states (i.e. waking (W), SWS and PS) also display major differences in spectral components of their EEG (Fig. 2). The EEG of SWS is characterized by oscillations of high amplitude and low frequencies. Power spectrum analysis of this EEG indicates a large predominance of low frequencies, including sleep spindles (8–14 Hz), delta waves (1–4.5 Hz, also defined as Slow Wave Activity (SWA)) and slow waves (<1 Hz), corresponding to a high level of synchronization of cortical cells discharge. More precisely, slow waves oscillations are composed by the alternation of synchronized cell firing periods (UP states) and silent periods (DOWN states) (Fig. 2b). While the muscle tone is lower in PS than during waking, the EEG signal is apparently close to the waking one. EEG displays oscillations of low amplitude and high frequencies. Power spectrum of the PS EEG is shifted towards more rapid frequencies with a specific peak at 5-7 Hz (theta band). This peak is mainly due to the firing rate rhythm of hippocampal neurons and is particularly apparent in rodents where hippocampus occupies a dorsal location. During waking, oscillations of low amplitude and rapid frequencies which correspond to desynchronization of cortical cells firing, are observed on the EEG. Depending animal behavior, EEG power spectrum during waking displays a relative increase in alpha (9-12 Hz), beta (12-30 Hz) and gamma (>30 Hz) frequency bands.

Alternation of SWS and PS (usually ended by a brief awakening) constitutes a sleep cycle whose duration is specific of each animal species. Moreover, in several mammals such as human, SWS can be subdivided in light-SWS and deep-SWS. Mammals display a great diversity in their total sleep time per day as well as in the PS quantity (Siegel 2001). For example, the big brown bat sleeps almost 20 h/day with 4 h in PS while a horse sleeps about 3 h/day with 0.5 h in PS. Such a difference in time spent asleep has been initially found positively correlated to metabolic rate and negatively correlated to brain size (Zepelin and Rechtschaffen 1974). However, more precise analysis found that sleep amount better reflects ecological constraints in term of foraging or risk of predation (Capellini et al. 2008). The daily sleep distribution, which also varies greatly across mammals, is likely also under the same ecological pressure and remains a major difference between man and other mammals classically used (rodents) in sleep research.

The regulation of sleep, although not totally understood, can be considered as the interaction between circadian clock and homeostatic mechanisms (Borbely and Achermann 1999). The molecular components (i.e. proteins) of circadian clock are present in each cell where their cyclic expressions are regulated by successive positive and negative transcriptional feedback loops (see Ko and Takahashi 2006; Panda et al. 2002). These cellular clocks are synchronized by the central clock of the body localized in the suprachiasmatic nucleus (SCN), a hypothalamic small nucleus situated just above the optic chiasma from which it directly receives inputs encoding light information (Dibner et al. 2010). In basal conditions, sleep periods occur with a circadian rhythmic pattern which is abolished when SCN is destroyed (for review see Moore 2007). Moreover, in humans and mice, polymorphisms for clock genes are correlated to different sleep-wake cycle disturbances such as the familial advanced sleep-phase syndrome (Per2 mutation) or familial delayed sleep phase syndrome (Per3 mutations) (Archer et al. 2010; Chong et al. 2012).

Sleep is also regulated via homeostatic mechanisms. Indeed, total sleep length increases following prolonged wakefulness; a phenomenon also called “sleep rebound”. Moreover, during SWS, an increase in SWA (Fig. 2a), corresponding to the power spectrum of the EEG delta band, is observed during the first hours of the recovery period subsequent to sleep loss. This SWA increase is generally considered as the most reproducible index of the homeostatic sleep regulation (Borbely et al. 1981; Borbely et al. 1984). Interestingly, PS also displays a homeostatic regulation which seems to be only reflected by an increase in length of its epochs without major change in the EEG power spectrum. During the last decade, experimental data in human and rodents showed that, during SWS, the SWA is greater in cortical areas which were more activated during the previous wakefulness period even in absence of sleep loss (Huber et al. 2004; Vyazovskiy et al. 2000). More recently, using intra-cortical recordings, Vyazovskiy and coworkers reported that electrophysiological features of the SWS (e.g. slow waves between 0.1-1 Hz) are also expressed by an increasing number of neurons in parallel to the waking duration (Vyazovskiy et al. 2011). These results indicate that the homeostatic regulation of SWA is a “local and use-dependent” phenomenon and challenge the classical view of sleep as the result of interactions between distributed excitatory and inhibitory neuronal networks (mainly in diencephalon and in rhombencephalon) (Krueger and Tononi 2011; Rattenborg et al. 2012).

## Benington and Heller hypothesis (BHH)

### Sleep as a state of brain energy conservation

Although the energy cost of each sleep state at cellular or sub-cellular levels (synapses) is not known, the cerebral variations of the energy consumption evaluated by glucose uptake measurements performed in human (Buchsbaum et al. 1989; Heiss et al. 1985; Nofzinger et al. 2002), monkey (Kennedy et al. 1982), cat (Ramm and Frost 1986) and rodents (Jay et al. 1985; Vyazovskiy et al. 2008) clearly indicate that SWS is an energy sparing stage relative to waking and PS. Measurements of regional cerebral blood flow (Braun et al. 1997; Madsen et al. 1991) and metabolic rate (Katayose et al. 2009) during sleep in human corroborate these results. These data led to hypothesize that one of the functions of sleep is to preserve brain energy levels, a fact that could be used to support the cellular mechanisms related to brain plasticity, especially those involved in off-line memory processes during sleep (Tononi and Cirelli 2006).

### Sleep and glycogen

In a pioneering study, Karadzic and Mrsulja in 1969 showed that 72 h of selective PS deprivation, in rats induce changes in glycogen content in different brain areas (Karadzic and Mrsulja 1969). These results therefore suggested a direct link between specific vigilance state (PS) and brain glycogen content.

Glycogen is classically thought to supplement glucose uptake by brain during physiological stimulation. A decrease in glycogen levels was observed in primary sensory cortical areas following sensory stimulation (Dienel and Cruz 2004; Swanson et al. 1992) and histochemistry of the GP (active a-form) has been used as metabolic marker of neuronal activation (Harley and Bielajew 1992; Woolf et al. 1985).

### The BHH

Consistent with this view, Karnovsky and co-workers conducted a pivotal study where they measured glycogen levels in rat brain following different times spent awake or different times spent asleep (Karnovsky et al. 1983). Their results showed that glycogen levels were greatly increased (+70 %) during the first 15 min of a sleep period whereas they decrease more slowly (−30 %) 20 min following an awakening. For instance, in vitro data indicate that neurotransmitters known to be specifically released during waking such as NA, serotonin and histamine (reviewed in Jones 2005), induce a rapid glycogenolysis (Magistretti et al. 1993; Quach et al. 1978; Sorg and Magistretti 1991). In addition, brain glycogen levels are lower in the first half of the dark period, when the activity of mice is maximal, and are higher during the rest period (Hutchins and Rogers 1970). Therefore, during activity period, repetitive glycogen degradation should occur in response to the different behavioral sequences and then, lead to a global glycogen depletion at the cortical levels. Because glycogen depletion should correspond to a decrease in ATP/AMP ratio, Ade levels should increase resulting of 5′-AMP degradation by ectonucleotidases. Ade could act on different types of receptors (mainly A1 and A2_A,_ A2_B_) present on neuronal cells. A1 receptor stimulation inhibits synaptic transmission through an increase in K + conductance (gK+) whereas A2_A_ receptor stimulation activates adenylyl cyclase and exhibits more vasodilatation and metabolic effects (for review see Sheth et al. 2014).

**Fig. 3:**
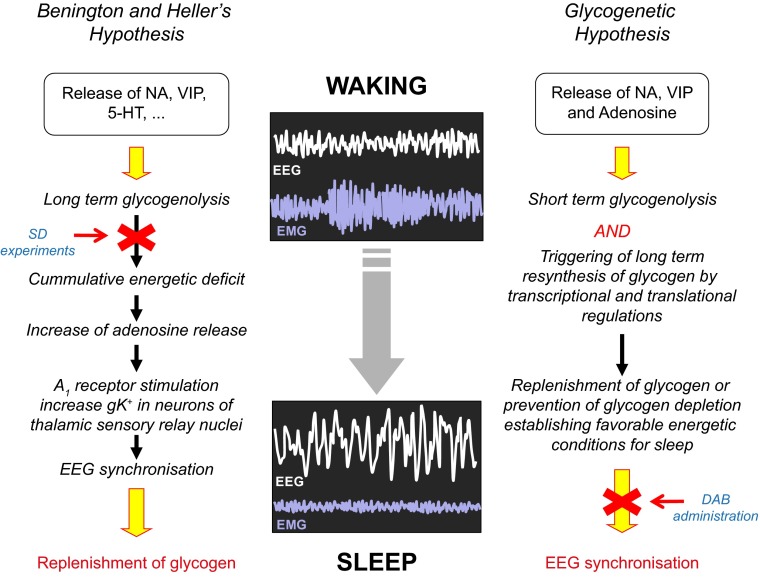
Energy hypotheses of sleep proposing glycogen as key regulator of sleep homeostasis. Schematic views of Benington and Heller’s hypothesis (*left side*) and the “glycogenetic” hypothesis (*right side*) are shown (see text for detailed description). Red arrows indicate steps that are not supported by experimental data. *NA*: noradrenaline; *VIP*: vasoactive intestinal peptide; 5-HT: 5-hydroxytryptamine or serotonin; *SD*: sleep deprivation; *DAB*: 1,4-dideoxy-1,4-imino-d-arabinitol; *EEG*: electroencephalogram; *EMG*: electromyogram

In summary, Benington and Heller’s hypothesis (BHH) proposed that in response to local increase in neuronal activity, the depletion of glycogen during waking induces a decrease in the ATP/AMP ratio resulting in accumulation of extracellular Ade. Extracellular Ade decreases the neuronal firing rate through its action on A1 receptor. Finally, the inhibitory action of Ade triggers sleep. Sleep, in turn, allows replenishing glycogen stores which create the beneficial energetic conditions for subsequent waking period (Fig. 3).

As a direct correlate of BHH, prolonged wakefulness should induce more profound glycogen depletion compared to undisturbed animals. Therefore, in order to obtain a direct demonstration of BHH different research groups assessed glycogen levels following acute total sleep deprivation (SD) in various species. One should note that the SD method used in the subsequent described studies, if not otherwise specified, is the so-called “gentle” sleep deprivation method. This SD method consists on gently modifying the animal environment (i.e. changing a part of its bedding material, introducing new object into the cage) when animal presents behavioral signs of sleepiness (i.e. prolonged immobility or adopting sleep posture).

### Testing the BHH

A first set of experiments was conducted by Heller’s group in which glycogen levels were measured in different brain regions following 6 h and 12 h periods of SD in young rats (Gip et al. 2002). In this study, animals were sacrificed using microwave irradiation (Gip et al. 2002) to preserve glycogen from rapid degradation as induced by killing animals by decapitation. Indeed, this method of sacrifice represents the best available method to measure true physiological concentrations of energy metabolites such as glycogen, lactate or glucose that display very rapid changes when ATP/AMP ratio falls. Results of this study indicate that cerebellar levels of glycogen were decreased after 6 h of SD whereas no change was observed in cerebral cortex. Interestingly, a decrease in both structures was only observed after 12 h of SD (Gip et al. 2002). Similar results were also obtained by Kong and co-workers in rats also sacrificed with a microwave irradiator (Kong et al. 2002). These authors, who measured glycogen in the whole brain (minus cerebellum and brainstem), showed that glycogen levels were decreased by 38 % after 12 h of SD.

**Fig. 4:**
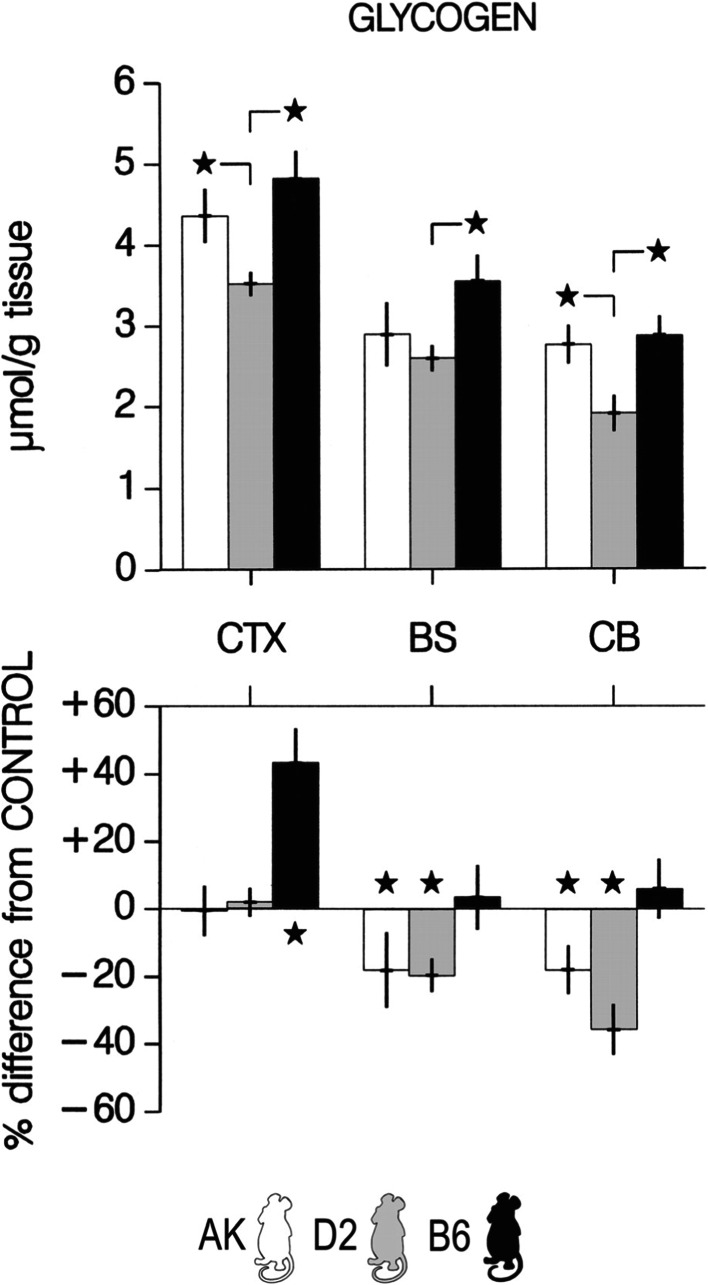
Changes in brain glycogen after sleep deprivation vary with genotype. Brain glycogen content in sleep-deprived mice. Brain glycogen varied with strain and structure (2-way ANOVA with repeated measures for factor structure. Factor strain, *F*_2,30_ = 13.2, *P* < 0.0001; structure, *F*_2,60_ = 71.9, *P* < 0.0001; interaction, *F*_4,60_ = 1.6, *P* = 0.20). Bars represent mean values ±2SE. *Top panels*: ^*^significant strain differences for each brain region (Tukey’s range test; *P* < 0.05). *Bottom panels*: ^*^significant differences from control (unpaired 2-sided *t*-tests; *P* < 0.05). In the *bottom panels*, values were expressed as % difference from the mean values in the control group (0 %). CTX, cerebral cortex; BS, brainstem; CB, cerebellum. Adapted from (Franken et al. 2003) with permission

The BHH has also been assessed in different inbred strains of mice (C57BL/6j (B6), AKR/j (AK) and DBA/2j (D2)) that differ in their homeostatic response in SWA to a 6 h of SD with the most pronounced response in AK mice and the lowest in D2 mice (Franken et al. 2003). Increased SWA response is thought to be correlated with more pronounced neuronal activity during the previous waking period. According to the BHH, this should consequently lead a larger glycogen depletion in AK mice than in D2 mice. Similarly to experiments performed in rats by Heller’s group (see above), Franken and co-workers sacrificed the mice after 6 h of SD by microwave irradiation and measured glycogen levels in the cerebral cortex, the brainstem and the cerebellum. The main results of this study indicate that glycogen levels increased by 40 % in the cortex of B6 mice whereas they remained unchanged in AK and D2 mice (Fig. 4). A straightforward conclusion from these observations is that mice that differ the most in their SWA response to SD did not exhibit differences in their glycogen levels. This absence of correlation therefore does not support BHH and suggests that factors other than homeostatic response in SWA may underlie strain differences in term of glycogen response to SD. For instance, it was proposed by the authors that differences in stress responses may govern such metabolic response (see below). In addition to this, these studies demonstrated that cerebral glycogen regulation displays some specific variations which depend on the brain region studied. Indeed, in contrast to what was found at the cortical level, glycogen levels decreased by 20-38 % in brainstem and cerebellum in AK and D2 mice though no change was observed in B6 mice (Fig. 4).

Impact of SD on brain glycogen levels has also been evaluated in the fruitfly (*Drosophila melanogaster*) (Zimmerman et al. 2004). In these experiments, Zimmerman and collaborators measured glycogen levels following 3 h or 6 h of rest deprivation (RD) (Zimmerman et al. 2004). While a decrease was observed in the whole head and body, 6 h of RD failed to induce a significant decrease in glycogen levels in the brain. Unexpectedly, it was also observed that brain glycogen levels decreased after a shorter RD (3 h).

As a whole, these observations pinpoint the absence of glycogen depletion in the cortex following a 6 h period of SD (as observed in rats, mice and drosophila) and therefore failed to validate BHH.

## The “glycogenetic” hypothesis

### Setting the context

Based on BHH, Magistretti and Borbèly later on proposed an alternative hypothesis linking sleep and glycogen metabolism in the brain, that we will call the “glycogenetic” hypothesis. This hypothesis is essentially based on in vitro results obtained in Magistretti’s laboratory on glycogen metabolism regulation using primary culture of astrocytes from postnatal mice. As opposed to BHH which only considers short term glycogenolysis effects (in minutes) induced by the neurotransmitters released during waking, the “glycogenetic” hypothesis relies on the observations that some of these same neurotransmitters (NA, VIP, Ade) also trigger long-term glycogen resynthesis (in hours) (Sorg and Magistretti 1992) (see above and Fig. 1b). As previously mentioned, this resynthesis involved induction of transcription factors (Cardinaux and Magistretti 1996) as well as transcription and translation of proteins involved in glycogen synthesis such as PTG which set glycogen in a synthetic mode (Allaman et al. 2000; Allaman et al. 2003; Berman et al. 1998; Printen et al. 1997). Hence, at the beginning of the waking period, one can postulate that release of these aforementioned neurotransmitters induces a net consumption of glycogen but, in parallel, also triggers a “glycogenetic program” leading to a delayed resynthesis of glycogen that will occur during wakefulness. As a matter of fact, such a “rebound” in glycogen levels was also observed in vivo after a single hypoglycemic episode (Choi et al. 2003) and in mice injected with amphetamine (Hutchins and Rogers 1970). Such glycogen regulation is also consistent with the observations made by Hutchins and Rogers (1970) which indicate that, while brain glycogen levels are low at the beginning of the active period, they increase before the end of this period (see Fig. 3 in Hutchins and Rogers 1970).

### The “glycogenetic” hypothesis

Actually, this alternative to BHH proposed that during wakefulness, in addition to their glycogenolytic effects, NA, VIP and Ade also trigger glycogen synthesis through transcriptional and translational effects. Therefore, glycogen levels should be maintained and even increased at the end of the wakefulness period (Fig. 3). Since the mechanisms which underlie this assumption have been obtained from cortical cells and since EEG signal reflected cortical activity pattern, the cerebral cortex was mainly considered as “the site” for “glycogenetic” hypothesis. In addition, as a consequence of the “glycogenetic” hypothesis and in contrast to the BHH we also proposed that glycogen preservation could have a role in the triggering of sleep and/or more particularly, in the occurrence and/or length of the PS episodes. As a matter of fact, PS is an energy consuming state that occurs following SWS episodes in which glucose consumption is low in the cortex. Thus, glycogen preserved during waking could be the cellular source of glucose required to ensure the neuro-metabolic coupling in response to increased neuronal activity during PS episodes (Fig. 3).

### Testing the “glycogenetic” hypothesis

In order to test this hypothesis, we first assessed the expression in the cerebral cortex of important genes encoding proteins related to glycogen metabolism such as GS, GP and PTG at four time points across the sleep-wake cycle as well as the GS activity following 6 h of SD in OF1 mice (Petit et al. 2002). Contrary to what was postulated, it was first observed that GS and GP mRNA levels were minimum during the active period and maximum during the rest period and that both decreased after 6 h of SD. However, PTG mRNA levels reached a maximum at the end of the active period and were markedly induced by SD (+94 % *vs* control value). Since PTG mRNA induction is known to promote GS activity and glycogen synthesis, one can logically deduce that a similar mechanism is engaged by 6 h of SD. Consistent with this, it was demonstrated that the active form of GS was significantly increased following SD in the cortex whereas the total form of GS (a + b forms) remained unchanged. Moreover, if ones assume that such a glycogenetic mode is taking place at the cortical levels this could provide an explanation for the absence of glycogen depletion at the cortical level following 6 h of SD as described in several studies (see above) (Franken et al. 2003; Gip et al. 2002; Zimmerman et al. 2004).

Even though the “gentle” SD method used in the previously described reports avoids stressful stimulations (acute intense noise or light, water or air puffs), plasma GCs levels are increased by two to fourfold in such protocols (Gip et al. 2004; Petit et al. 2010; Tobler et al. 1983).

In order to establish whether or not these metabolic changes were restricted to the “gentle” SD method, we also explored the impact of a pharmacological SD, which does not require intervention and therefore limits possible stress confounding effects. To this end we used modafinil (MOD) which is a non-amphetamine vigilance–promoting drug classically used in narcoleptic patients (Wise et al. 2007). In mice, a single injection of this drug induces a continuous wakefulness during 5–6 h followed by a SWA rebound in the following period similar to those obtained after gentle SD (Kopp et al. 2002). Different glycogen metabolism indices including levels of mRNA encoding GS, GP and PTG as well as glycogen levels were measured in the cortex of mice. Whatever the method used for SD (gentle or pharmacological), it was observed that cortical PTG mRNA levels and GS activity were consistently increased (Petit et al. 2010). In addition, we showed that glycogen levels displayed no change in the cortex in both SD methods used, indicating that glycogen regulation is driven by the prolongation of wakefulness and not by the “gentle SD” per se. Interestingly, PTG mRNA and glycogen levels were normalized following 3 h of sleep recovery. Induction of PTG mRNA levels was also reported in cDNA microarray analysis performed following SD experiments in mice (Maret et al. 2007; Mongrain et al. 2010). We should also notice that an induction of glycogenin, is also regulated by “gentle” SD, suggesting that *de novo* granules formation could also participate to glycogen regulation after SD (Petit et al. 2010). Along the same line, a single dose of methylphenidate (Ritalin™) which is able to maintain B6 mice awake for 3–4 h, also induced a significant increase in PTG mRNA levels (+50 %) as well as in glycogenin mRNA (+22 %) in the anterior part of the cortex (unpublished data).

These results argue in favor of a glycogen synthesis occurring during waking in parallel to glycogenolysis induced by repeated sensory-motor stimulations or pharmacological neuronal stimulations. This is likely achieved by the increase in PTG levels which is able to direct glycogen metabolism preferentially towards synthesis.

### Cortical glycogen turnover increases during prolonged wakefulness

One of the limitations of determination of glycogen levels at the end of a SD, or following sleep recovery, is that it doesn’t allow the dynamic measurements of glycogen metabolism during these periods. By measuring the incorporation of [1-^13^C]glucose into glycogen by Nuclear Magnetic Resonance (NMR) spectroscopy, Gruetter and co-workers determined glycogen concentration and turnover in rat brain in vivo (Choi et al. 1999; Choi and Gruetter 2003; Lei et al. 2007). This method has been used to determine glycogen concentration and turnover in euglycemic rats maintained awake during 5 h at the beginning of the rest period, compared to undisturbed rats (Morgenthaler et al. 2009). Following normalization by the N-acetyl-aspartate turnover, that remains constant, it was demonstrated that glycogen cycles faster (2.9 *vs* 5.3 h, *p* < 0.05) in rats continuously maintained awake by sensory stimulations during the labeling period, revealing an increase in glycogen turnover induced during prolonged wakefulness. In contrast, glycogen concentration remained unchanged in the same animals (4.0 *vs* 3.9 μmol/g) (Morgenthaler et al. 2009). These results are in accordance with those obtained in rodents and flies following 6 h of SD and with our results showing an increase in GS activity in spite of constant glycogenolysis evoked by prolonged wakefulness (Petit et al. 2010).

## A synthesis of experimental data

Based on these experimental data, we can propose the following model of glycogen regulation throughout the sleep-wake cycle:

- When arousal occurs, the rise in wakefulness-related neurotransmitters (monoamines, VIP and Ade) (e.g. see Takahashi et al. 2010) triggers a rapid (within few minutes) glycogenolysis throughout the whole cortex. This phase corresponds to the fall in glycogen concentration after 2–5 min of spontaneous awakening reported by Karnovsky and collaborators (Karnovsky et al. 1983). During the waking period, glycogenolysis is also repeatedly triggered in specific areas corresponding to sensory modalities solicited by novel environmental conditions and in motor areas. This view is in accordance with an important neuromodulatory role of the cortical projections of the noradrenergic cells from the locus coeruleus. These cells, in addition to a tonic mode of discharge during waking, display a phasic firing in response to salient sensory stimuli (Berridge and Waterhouse 2003; but see also Foote et al. 1983).
- At the same time, NA (and probably VIP and Ade) triggers the transcription and translation of PTG leading to an increase in GS activity.
- Therefore, a balance between synthesis and degradation progressively settles during the activity period and prevents glycogen decrease. This balance is also dependent of the stress levels since GCs favor glycogenolysis (see below). It appears that, this relative “steady state” cannot be maintained when wakefulness duration is prolonged more than 6–10 h in rodents as suggested by the results obtained after 12 h of SD (Gip et al. 2002; Kong et al. 2002).
- When sleep emerges, the inhibition of the sensory inputs to the cortex together with the decrease in the monoaminergic tone, reduces the cortical energy needs and consequently decreases glycogen consumption. This rapid unbalance in support of glycogen synthesis could explain the large increase in glycogen levels observed by Karnovsky and collaborators 15 min after sleep onset (Karnovsky et al. 1983).
- Finally, PTG levels and glycogen synthesis diminish during sleep as indicated by glycogen levels measured following 3 h of sleep recovery (Petit et al. 2010).

## Effect of direct modulation of glycogen levels on sleep

Because experimental data support the glycogen maintenance proposed by the “glycogenetic” hypothesis, we wanted to test a physiological consequence of this observation which assumes that glycogen levels per se could regulate sleep. To investigate this aspect we took advantage of pharmacological agents that block glycogen degradation (Treadway et al. 2001).

### 1,4-dideoxy-1,4-D-arabinitol (DAB) as pharmacological tool

In order to normalize glycemia, a large number of molecules inhibiting GP through different binding sites were designed (Oikonomakos and Somsak 2008). Among them, several glucose analogs based on iminosugars such as Isofagomine or DAB act on the active form of GP (GP a-form), the rate-limiting enzyme of glycogen mobilization, by allosteric inhibition (Somsak et al. 2003). In vitro, DAB potently inhibits GP in different preparations (Andersen and Westergaard 2002; Somsak et al. 2003) including brain tissue homogenates (IC_50_ ≈ 0.4 μM) (Walls et al. 2008). Moreover, DAB is effective in blocking glycogen mobilization in astrocytes as shown by the full prevention of NA-induced glycogenolysis by this compound in astrocytes in culture (Walls et al. 2008). Importantly, DAB was used to demonstrate the importance of brain glycogen mobilization for the establishment of memory formation (Gibbs et al. 2006; Newman et al. 2011; Suzuki et al. 2011). The last set of results confirms DAB as a relevant pharmacological tool to investigate glycogen function in vivo.

### Testing the blockade of glycogen in spontaneous sleep-wake cycle

**Fig. 5:**
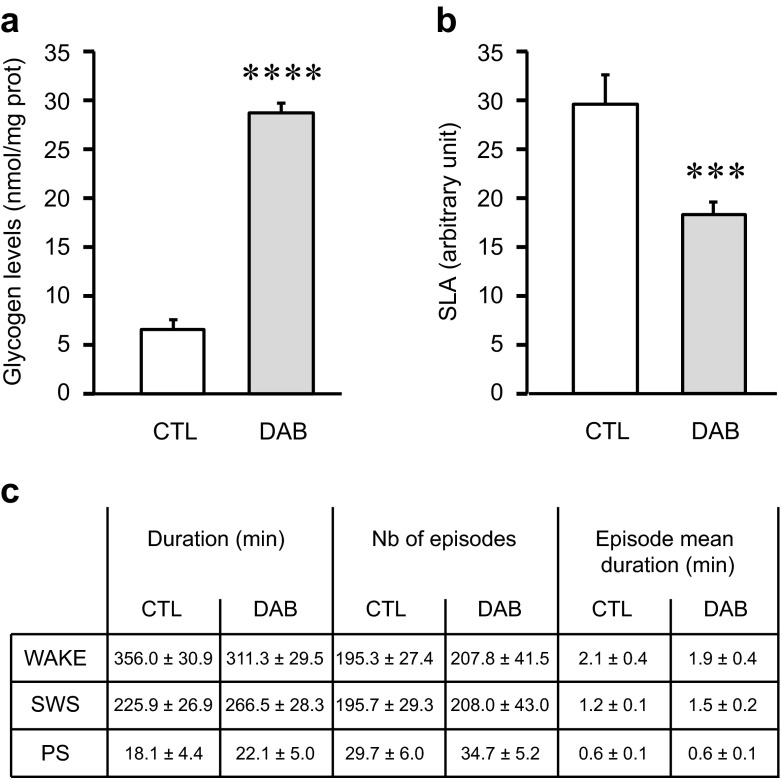
Effects of acute pharmacological inhibition of glycogen mobilization on locomotor activity and sleep parameters when added at dark onset. Experiments were performed on adult males mice (C57BL/6j from Janvier, France) equipped with EEG and EMG electrodes (for further details see Petit et al. 2013) and implanted with a chronic cannula into the lateral ventricle. The mice were housed individually under a 12-h:12-h light–dark cycle at 23 °C ambient temperature with food and water ad libitum. Two weeks after the surgery, they were injected with 2 μl of NaCl 0.9 % (control [CTL] group, white bars) or 1,4-dideoxy-1,4-imino-D-arabinitol (DAB) at 0.5 M dissolved in NaCl 0.9 % (DAB group, gray bars) at the beginning of the dark period (Zeitgeber Time 12, ZT12) under a light isoflurane anesthesia. After injections, mice were let 2 h in their cages for anesthesia recovery before recordings begin. **a** Brain glycogen levels 3 h after i.c.v. administration of DAB. After the injection, the animals were replaced in their cages and sacrificed 3 h later (ZT15). The brains were quickly removed, frozen and cut for site injection verification and standard glycogen dosage (Petit et al. 2010). The values represent the mean of glycogen levels in nmol/mg prot (±SEM) measured on brain slices from -200 μm posterior to +200 μm anterior compared to the injection site in both group (CTL vs DAB). *N* = 6. Statistics: Unpaired *t* test with Welch’s correction,**** *p* value <0.0001 compared to CTL. **b** Spontaneous Locomotor Activity (SLA) after i.c.v injection of DAB. Each cage was equipped with passive infrared sensors on the top for SLA recordings. SLA was recorded for 10-min interval during 10 h from ZT14 to ZT24. The same animals received NaCl 0.9 % and DAB on separate days. The values represent mean SLA ± SEM over this 10 h period in both group (CTL vs DAB). *N* = 12. Statistics: Paired *t* test, *** *p* value = 0.0004 compared to CTL. **c** Quantitative parameters of the sleep wake cycle after i.c.v. injection of DAB. The EEG/EMG signals were recorded and digitalized with an Embla A10 amplifier (Medcare, USA). The EEG was divided in 4-s epochs which were visually scored in one of the three states of vigilance (wakefulness (W), slow wave sleep (SWS) or paradoxical sleep (PS)) according to classical criteria (Tobler et al. 1997). Values represent the mean values (±SEM) of cumulative duration (min), number of episodes and episode mean duration (min) for each state of vigilance for the 10 h of recording session from ZT14 to ZT24. *N* = 6 animals in each group. Statistics: One way ANOVA followed by Bonferroni’s post-test. No statistical difference was observed between the control and the DAB groups

In order to assess more directly the role of glycogen on the sleep-wake architecture and /or on the occurrence and the duration of PS epochs, we used DAB to block brain glycogen mobilization in mice housed in basal conditions (light–dark cycle: 12:12), (Fig. 5). Mice received an intra-cerebro-ventricular (i.c.v) injection of either saline solution (control) or DAB (0.5 M). Each injection was performed at the light/dark shift (i.e. zeitgeber time (ZT) 12) that corresponds to the beginning of the active period in rodents.

We first verified the GP inhibitor efficiency in vivo by measuring cortical glycogen levels 3 h after i.c.v injection of DAB. As expected, glycogen levels were significantly more elevated (fivefold) in the DAB group as compared to control group (Fig. 5a).

In a second set of experiments, using a similar protocol, we assessed a possible effect of glycogen mobilization blockade on spontaneous locomotor activity (SLA) in mice. Injections were also performed at ZT12 and SLA was continuously recorded during 10 h from ZT14 until the end of the dark period (ZT24). During this period, SLA was significantly decreased by 38 % in DAB mice as compared to control (Fig. 5b). This last result suggests that an inhibition of glycogen mobilization in the brain might induce an increase of sleep and/or quiet wake in mice.

To clarify this last point, we repeated the experiment with mice already implanted with EEG/EMG electrodes and wire-connected for sleep recordings. DAB or saline were injected at ZT12 and classical vigilance states parameters were recorded over a 10 h period (ZT14-ZT24). Results did not indicate any significant effect of DAB on SWS nor on PS parameters during this recording period (Fig. 5c). To investigate whether GP inhibition could alter sleep more qualitatively than quantitatively, we also performed a spectral analysis of the EEG during SWS epochs (by 2 h bin from ZT14 to ZT 24 in control and DAB-injected mice). For this, we focused our analysis on the SWA (power spectrum of the delta frequency band) which represents an index of sleep depth. A slight but non-significant decrease in SWA was observed following DAB injections indicating that the depth of the SWS was not altered by the glycogen blockade (data not shown).

These last results indicate that the large decrease in locomotor activity observed after DAB injection is not due to increase in sleep duration, even though an increase of quiet wake/drowsiness could not be excluded. This could suggest that blockade of glycogen mobilization might impact more specifically brain areas involved in locomotion. Similarly, intraperitoneal injection of caffeine at a dose (200 mg/kg) that inhibits GP, also depressed the locomotor activity in mice (Hutchins and Rogers 1970). Thus, and in contrast to the “second part” of our hypothesis where we proposed that maintaining glycogen levels during waking could regulate sleep, acute blockade of glycogen mobilization by DAB has no effect on sleep parameters during the subsequent active period. In particular, our results cast some doubt on the possibility that glycogen could be used as energy source for PS episodes since no change in both number or length of PS epochs was observed. Moreover, our DAB experiments constitute an additional argument that doesn’t support the BHH. Indeed, according to this assumption, glycogen mobilization blockade should create energy deficits more rapidly and, consequently, should increase length and/or number of sleep episodes for activity period, a fact that is not verified experimentally (see above).

Based on this study with DAB, sleep occurrence and duration appear to be relatively independent to brain glycogen content, at least on a relative short period (about 10–12 h) and in standard environment.

However, it cannot be excluded from this study that glycogen mobilization blockade affects other cellular processes in sub-cortical structures such as hippocampus where memory mechanisms occur (for example the sharp waves ripples) during sleep stages (for review see Rasch and Born 2013).

## Confounding effect of GCs

### GCs affect cerebral metabolism

GCs are adrenal steroid hormones released in response to stressors. As previously mentioned, stress is a possible confounding factor that accompanies most standard SD protocol (which can result in an increase of plasma corticosterone, a physiological stress marker in rodents i.e. the main GCs in rat and mice).

An important role of GC in peripheral tissues is the regulation of glucose homeostasis (hence their name) via, among others, effects on glycogen metabolism (for review see McMahon et al. 1988). Although some direct effects of these steroid hormones on glycogen metabolism have been described, it is well recognized that they also act indirectly by influencing cells’ responsiveness to other hormones (Sapolsky et al. 2000). For example, it was demonstrated that, in perfused liver of adrenalectomized rats, stimulation of glycogenolysis by adrenaline and glucagon was reduced compared to control animals (Exton et al. 1972).

### GCs and sleep deprivation

GCs affect cerebral metabolism by modulating glucose uptake and utilization in cultured neurons and astrocytes (Allaman et al. 2004; Horner et al. 1990; Landgraf et al. 1978; Virgin et al. 1991). Adrenalectomy is followed by decreased glucose levels and increased glycolytic intermediates in the cortex (Plaschke et al. 1996). In most brain regions, GCs decrease local cerebral glucose utilization, whereas adrenalectomy increases it (e.g. see Doyle et al. 1994; Kadekaro et al. 1988). GCs also affect brain glycogen stores. In cultured cortical and hippocampal astrocytes, GCs decrease glycogen levels (Allaman et al. 2004; Tombaugh et al. 1992). Similarly, adrenalectomy can either increase brain glycogen levels (Passonneau et al. 1971) or have no effect in the whole brain (Goldberg and O’Toole 1969) or in the cortex (Plaschke et al. 1996).

Because GCs influence brain glycogen levels and are increased during SD, this raises the question of the contribution of GCs to changes of glycogen levels observed during SD as well as for their role in the homeostatic regulation of sleep. A first observation suggesting a link between glycogen metabolism and GCs during SD has been obtained by Heller and collaborators (Franken et al. 2003). Using three inbred mice strains, i.e. AK, D2, and B6, they showed that changes in brain glycogen after SD are strongly influenced by brain region and genotype (see above and Fig. 4). Although corticosterone levels after SD were not measured in this work, it was suggested that genotype differences in corticosterone induction during SD might contribute to the glycogen increase observed in B6 mice and the decreases observed in AK and D2 mice, based on studies showing a decreased corticosterone response to stress in B6 mice (e.g. see Roberts et al. 1992; Ryabinin et al. 1999).

Heller’s group investigated the influence of GC during SD by measuring glycogen stores in different brain regions in intact and adrenalectomized (with corticosterone replacement; Adx) Long-Evans rats after 6 h of SD (Gip et al. 2004). Following SD in control animals, glycogen levels decreased in the cerebellum and hippocampus but not in the cortex or brainstem. In contrast, glycogen levels in the cortex of Adx rats increased by 43 % after SD, while other regions were unaffected. These results unequivocally demonstrate that in the absence of a GC surge (prevented by adrenalectomy), glycogen stores in all investigated brain regions were spared, implying a direct effect of GCs on cerebral glycogen levels regulation during SD. Moreover, this is in agreement with the notion that elevated GCs secretion during SD causes brain glycogenolysis and unbalances the synthesis/degradation ratio.

In addition to changes of glucose metabolism, the effect of adrenalectomy observed in Heller’s study on glycogen levels may be explained by in vitro data that showing a suppressing action of GC on the synthesis of glycogen induced by NA in astrocytes (Allaman et al. 2004). In Allaman et al. (2004) it was observed that exposure of primary cultures of cortical astrocytes to dexamethasone (DEX), a synthetic GC, results in the reduction of NA-induced glycogen resynthesis. DEX does not act through alteration of signal transduction mechanisms, as cAMP formation or PTG mRNA induced by noradrenergic stimulation was unchanged. In this context, it would be of interest to determine whether the suppressing action of GCs on long-term glycogen regulation by NA is selective or also effective for other neuromodulators such as VIP and Ade. These observations also support the view that glycogen metabolism during SD may be balanced between synthesis (e.g. induced by NA and Ade) and degradation (induced by GCs per se but also by the suppressive effects on NA-induced glycogen synthesis) which may increase glycogen turnover. Such view is strengthened by the observations that prolonged wakefulness increases glycogen turnover in the cortex as shown by a NMR study (see above) (Morgenthaler et al. 2009).

One should note that in the experiments by Heller’s and collaborators on Adx Long-Evans rats, SD protocol induces a massive increase in GCs in intact animals (at least 8 fold increase) (Gip et al. 2004). For comparison, the GCs fold-increase observed in AK and B6 mice are around 2 and 3, respectively (8 for D2, which is similar to the value for Long-Evans rats in Gip et al. 2004). Therefore, the high amplitude in GCs surge during SD as observed in Long-Evans rats may also amplify (override normal regulation) the GCs effects on glycogen levels. Interestingly, and as mentioned above, Petit et al (2010) compared the effects of wakefulness induced by “gentle” SD and or following injection of the wakefulness-promoting drug MOD on the expression of genes related to glucose and glycogen metabolism, GS activity as well as on glycogen levels in the cortex of the outbred mice strain OF1 (Petit et al. 2010). In this study plasma corticosterone were not influenced by MOD whereas a moderate (2 fold) increase was observed following “gentle” SD. Despite this differential effect on GCs plasma levels, MOD and “gentle” SD exerted a similar influence on gene expression including PTG, GS activity and on glycogen levels. It could thus be concluded that the mild stress induced by “gentle” SD was unlikely involved in the effects of “gentle” SD on glycogen metabolism indices analyzed including glycogen content at the cortical level.

### Are GCs modulators of sleep homeostasis?

An important question is therefore the role of GCs surge in the homeostatic regulation of sleep. In human, plasma cortisol (and corticosterone in rodents) concentration follows a circadian rhythm independent of sleep. It peaks at the time of awakening and slowly decreased during waking to reach a minimum when sleep begins (Van Cauter et al. 1991). This observation suggests a role of GCs in the building of sleep pressure. Recently, Mongrain and collaborators provided an important piece of work to answer this question (Mongrain et al. 2010). In this study, they aimed to elucidate the contribution of the corticosterone component of the stress response to the sleep-wake associated changes in the electrophysiological correlates of sleep need. To this end, they took advantage of D2 mice which display high corticosterone increase following SD to determine the contribution of this high corticosterone increase to blunted SWA rebound in Adx animals with corticosterone replacement. These results clearly demonstrated that while Adx successfully abolished the SD–induced increase in corticosterone secretion, Adx affected neither the baseline dynamics of SWA nor the increase in SWA after sleep deprivation as compared to in both intact controls and the sham-lesioned mice. Importantly, quantity and distribution of sleep, similarly to SWA during SWS, remain unaffected. The fact that Adx does not influence the SWA response to SD, argues against a major role of GCs in the homeostatic regulation of sleep. More importantly, whatever are the effects of GCs in SD in the regulation of glycogen levels, GCs do not appear to participate in the homeostatic regulations of sleep as shown by Mongrain’s work. Therefore, such results (modulation or absence of modulation of glycogen levels by GCs as observed in the different studies presented in this section) also questioned the importance of glycogen store replenishment as a regulator of sleep homeostasis.

## Concluding remarks

During the last decade, efforts were made to validate or invalidate the original hypothesis made by Benington and Heller on the role played by glycogen metabolism in the homeostatic regulation of sleep (part of these data have already been reviewed by Scharf and collaborators (Scharf et al. 2008)) . When considering results obtained in the different studies reviewed here, one can conclude that, contrary to what was initially postulated (i.e.glycogen reserves are mobilized during wakefulness), cortical glycogen levels remain relatively stable throughout the sleep-wake cycle, except at the beginning of rest and active periods, where glycogen levels rise and fall respectively for few minutes in response to rapid change in neuronal activity. More striking, glycogen reserves did not consistently decrease when wakefulness is prolonged.

Most results rather support the notion that, during wakefulness, a parallel glycogen synthesis and degradation sets resulting in a glycogen turnover increase. This is in accordance with the “glycogenetic” hypothesis which postulates the occurrence of a glycogen synthesis during waking. However, the assumption of the “glycogenetic” hypothesis that glycogen synthesized during waking could favor sleep (SWS and /or PS), was not verified in the experiments in which glycogen mobilization was pharmacologically blocked by DAB (Fig. 5), at least when it was acutely administrated.

Hence, none of the actual results (including GCs effect on sleep homeostasis) fully support a direct correlation between sleep and glycogen regulation (i.e. BHH or “glycogenetic” hypothesis) (Fig. 3). Nevertheless, a link between glycogen metabolism and sleep mechanisms could not be totally excluded. Indeed, experimental studies present several limitations that may impede the establishment of a direct role of glycogen metabolism in sleep regulation:

- Modulation (i.e. no change or increase) of glycogen levels was mainly assessed in the cortex (or whole brain) extracts of rodents whereas only few studies investigated possible changes in glycogen level in other brain areas such as cerebellum or brainstem. Interestingly, in these latter two brain areas a different regulation of glycogen content after SD was observed, suggesting that association between sleep homeostasis and glycogen levels may be stronger in these regions than in the cortex or whole brain extracts. Therefore, studies focusing on different and specialized brain structures might reveal important link between glycogen metabolism and sleep. In particular, comparison of sleep-induced glycogen levels variation between sleep-promoting areas (preoptic area, basal forebrain,…) and wake-promoting areas (posterior hypothalamus, locus coeruleus area,…), which may be supposed to display more pronounced variations during vigilance states, may bring interesting information to address this issue. Similarly, while glycogen-containing astrocytes can be found in both grey and white matters, only one study specifically assessed glycogen levels changes in white matter during SD (Kong et al. 2002). It was observed that SD impacted glycogen levels in both white and grey matters equally, indicating that white matter glycogen may also undergo specific regulation during sleep which may deserve particular interest.
- Regulation of glycogen metabolism is likely better reflected by turnover determinations rather than by static measurements of glycogen levels, as indicated by the work of Morgenthaler and collaborators using NMR spectroscopy (Morgenthaler et al. 2009). Therefore, efforts to establish associations between brain glycogen turnover and sleep homeostasis would be of interest in order to delineate the importance of glycogen metabolism in this process.
- It is classically assumed that SD is a stress per se, hence unavoidably accompanying SD protocols. While GCs do not constitute the only stress marker, an increase in GCs is usually observed in classical SD procedures (even in “gentle” sleep deprivation such as in most of the studies reviewed here (Gip et al. 2004; Petit et al. 2010)). Since, GCs affect glycogen regulation by dampening its synthesis, GCs have to be considered as a confounding factor in SD protocols. Fortunately, there are means to circumvent the GCs issue. For instance, pharmacological SD as used by Petit and co-workers illustrates such a possible way to avoid increase in plasma GCs (Petit et al. 2010). Moreover, the development of new non-stressful SD devices represents another interesting alternative way to prevent stress effects in metabolism-sleep interactions studies (e.g. see Leenaars et al. 2011; Petit et al. 2013).
- Mechanisms of glycogen regulation based on the “glycogenetic hypothesis” are supported by results obtained for 6 h SD. When wakefulness is more prolonged (such as in 12 h of SD), glycogen depletion is observed (Kong et al. 2002), suggesting that other mechanisms of glycogen regulation take place.

To conclude, we should underline that thanks to the initial BHH, several experiments have been performed and contributed to test it which have contributed a better understanding, albeit incomplete, of the relationship between glycogen regulation and sleep homeostasis. Even if the Benington and Heller’s assumption that sleep serves for glycogen replenishment is not supported by the experimental data, their hypothesis underlined the important role of astrocytic energy metabolism in sleep regulation. Quite recently, such a role of astrocytes related to Ade has been uncovered. For instance, Halassa and co-workers reported the loss of SWA rebound after SD in mice in which the vesicular release of ATP, the precursor of Ade, was suppressed specifically in astrocytes (Frank 2013; Halassa et al. 2009). In fact, a body of evidence indicates that astrocytes-released ATP modulates neuronal transmission by acting on A1 receptors which ultimately leads to a SWA increase (Blutstein and Haydon 2013). Interestingly, ATP release evoked by glutamate, potassium and adenosine in cultured astrocytes was shown to be prevented by DAB (Hertz et al. 2014), providing a possible yet unsuspected link between glycogen metabolism, ATP release and sleep regulation. In line with the importance of astrocytes in higher brain functions, the involvement of glycogen metabolism in memory processes has recently been unveiled in rodents (Newman et al. 2011; Suzuki et al. 2011).
